# Sex differences in workload-indexed blood pressure response and vascular function among professional athletes and their utility for clinical exercise testing

**DOI:** 10.1007/s00421-021-04656-x

**Published:** 2021-03-12

**Authors:** Pascal Bauer, Lutz Kraushaar, Oliver Dörr, Holger Nef, Christian W. Hamm, Astrid Most

**Affiliations:** 1 Department of Cardiology and Angiology, Justus- Liebig- University Giessen, Giessen, Germany; 2 Adiphea GmbH, Werbach, Germany; 3 Department of Cardiology, Kerckhoff Clinic GmbH, Bad Nauheim, Germany

**Keywords:** Exercise test, Professional athletes, Sex differences, Vascular function, Workload-indexed blood pressure response

## Abstract

**Purpose:**

Sex differences in blood pressure (BP) regulation at rest have been attributed to differences in vascular function. Further, arterial stiffness predicts an exaggerated blood pressure response to exercise (BPR) in healthy young adults. However, the relationship of vascular function to the workload-indexed BPR and potential sex differences in athletes are unknown.

**Methods:**

We examined 47 male (21.6 ± 1.7 years) and 25 female (21.1 ± 2 years) athletes in this single-center pilot study. We assessed vascular function at rest, including systolic blood pressure (SBP). Further, we determined the SBP/W slope, the SBP/MET slope, and the SBP/W ratio at peak exercise during cycling ergometry.

**Results:**

Male athletes had a lower central diastolic blood pressure (57 ± 9.5 vs. 67 ± 9.5 mmHg, *p* < 0.001) but a higher central pulse pressure (37 ± 6.5 vs. 29 ± 4.7 mmHg, *p* < 0.001), maximum SBP (202 ± 20 vs. 177 ± 15 mmHg, *p* < 0.001), and ΔSBP (78 ± 19 vs. 58 ± 14 mmHg, *p* < 0.001) than females. Total vascular resistance (1293 ± 318 vs. 1218 ± 341 dyn*s/cm^5^, *p* = 0.369), pulse wave velocity (6.2 ± 0.85 vs. 5.9 ± 0.58 m/s, *p* = 0.079), BP at rest (125 ± 10/76 ± 7 vs. 120 ± 11/73.5 ± 8 mmHg, *p* > 0.05), and the SBP/MET slope (5.7 ± 1.8 vs. 5.1 ± 1.6 mmHg/MET, *p* = 0.158) were not different. The SBP/W slope (0.34 ± 0.12 vs. 0.53 ± 0.19 mmHg/W) and the peak SBP/W ratio (0.61 ± 0.12 vs. 0.95 ± 0.17 mmHg/W) were markedly lower in males than in females (*p* < 0.001).

**Conclusion:**

Male athletes displayed a lower SBP/W slope and peak SBP/W ratio than females, whereas the SBP/MET slope was not different between the sexes. Vascular functional parameters were not able to predict the workload-indexed BPR in males and females.

## Introduction

There is a wealth of evidence indicating a different blood pressure (BP) regulation at rest between women and men (Briant et al. 2016; Song et al. 2020). Several physiological factors have been identified that influence these differences, including sympathetic nervous activity, renin-angiotensin system, β-adrenergic vasodilatation, peripheral vascular resistance, arterial stiffening and sex hormones (Briant et al. 2016; Smith et al. 2019; Song et al. 2020; Safar et al. 2020; Ramirez and Sullivan 2018; Hermida et al. 2013; Ochoa-Jimenez et al. 2018). However, the threshold for defining hypertension was set at the same level of BP for men and women in the current guidelines (Williams et al. 2018).

The clinical impact of the blood pressure response (BPR) during exercise is another controversial issue (Sabbahi et al. 2018; Percuku et al. 2019; Hedman et al. 2019). Despite the recommendations in former guidelines (Mancia et al. 2013), which proposed different BP thresholds to define an exaggerated BPR for males and females, the European Society of Cardiology states in its latest guideline that there is currently no consensus on normal BPR during exercise (Williams et al. 2018). Thus far, the sex differences in BP regulation during exercise are not well characterized (Hedman et al. 2020; Currie et al. 2018). Though, the blood pressure response to graded clinical exercise testing offers the potential to uncover occult cardiovascular (CV) pathology and future CV risk that may go undetected by routine office measurements at rest (Caselli et al. 2019). Functional vascular impairment might lead to an exaggerated BPR to exercise even in the absence of hypertension at rest (Miyai et al. 2020; Thanassoulis et al. 2012). Novel markers of vascular function have emerged whose feasibility for acquisition at-rest via noninvasive oscillometric devices could simplify clinical assessment in uncovering this functional impairment (Miyata 2018).

Recently, a workload-indexed approach of characterizing BPR (SBP/MET slope) was introduced (Hedman et al. 2019) for the general population and for athletes (Bauer et al. 2020). Considering that a higher SBP/MET slope (> 6.2 mmHg/MET) was associated with worse survival in a normal population of male non-athletes (Hedman et al. 2019), these data suggest that a steeper increase in SBP in relation to workload is a stronger prognostic factor of mortality in males than the peak SBP. Therefore, the proposed SBP/MET slope might be a useful tool to identify individuals at risk, which would be crucial for preventive interventions.

In addition, normative age- and sex-adjusted values for new workload-indexed BPR markers such as the SBP/W slope and the peak SBP/W ratio for the general population have been published (Hedman et al. 2020). The peak SBP/W ratio represents the ratio of peak SBP to maximum achieved W in response to bicycle ergometer, whereas the SBP/W slope reflects the increase of SBP per W increment and thus the steepness of SBP in relation to workload with higher values representing a steeper increase. Of note, the presented normative values were markedly higher in females than in males, indicating a steeper BP increase during exercise (Hedman et al. 2020). These sex differences in the BP regulation are thought to be modulated by vascular function (Song et al. 2020; Haarala et al. 2020; Ayer et al. 2010; Wee et al. 2019) and thus might be revealed by measuring vascular function and central hemodynamics at rest and under exercise conditions (Hedman et al. 2020).

Given the postulated sex differences in SBP/W slope, peak SBP/W ratio and the unknown effect of sex on the SBP/MET-slope, we investigated sex differences in these markers of workload-indexed BPR in age-matched professional athletes to aid physicians in interpreting the BPR to exercise in the cardiovascular evaluation of athletes. Further, we speculated that markers of arterial stiffness, like PWV, Aix@75 and peripheral resistance, could predict the workload-indexed BPR of both sexes. In addition, we investigated the association of central hemodynamics and vascular function with the recently introduced workload-indexed markers of the BPR to exercise.

## Materials and methods

### Study design

The study was conducted as a cross-sectional, single-center pilot study as part of the routine pre-season medical monitoring program of the first German handball and female soccer division in July 2019. Competitive team handball and competitive soccer are both classified as high-intensity mixed sports with a high load for the cardiovascular system. They are characterized by requiring the repetition of high-intensity activities with brief recovery periods. Players need the ability to perform repeated maximal or near maximal intensities such as sprinting, jumping and changing of directions throughout the match.

All participants received a clear explanation of the study and provided their written informed consent. Further, they filled out a questionnaire regarding health status, medication, nutrition supplementation, amount of training, and history of training (pre-participation questionnaire of the European Federation of Sports medicine associations). Only healthy individuals free of underlying cardiovascular diseases, risk factors, and medication (other than oral contraceptives) were included.

The examination took place at noon between 12:00 and 14:00 o’clock and was scheduled in the first week of the new season after a 6-week competition-free interval. The last time athletes had trained was 36 h prior to the study beginning; the last allowed meal was breakfast up to 3 h before the investigation. There was no restriction of caffeine intake provided. Thus, alcohol consumption was prohibited the two days prior to the study beginning. The day before the examination was filled with commercial dates with no physical effort.

The local ethics committee approved the study protocol. The study was performed in accordance with the ethical standards laid down in the Declaration of Helsinki and its later amendments. All subjects gave written informed consent to participate.

### Study population

The participants were 72 healthy professional athletes consisting of 47 male handball and 25 female soccer players. All participants included were Caucasian, non-smokers and none took medication (except oral contraceptives) or multivitamin supplements. All female athletes were examined during the early follicular phase or in the placebo phase for those taking oral contraceptives to minimize hormonal effects. As age was found to influence the BPR to exercise in both sexes, only athletes aged 18–24 were included in the study.

All individuals were subjected to a physical examination, 12-lead electrocardiogram (ECG), and progressive maximal cycling ergometer test. Age, height, weight, and body mass index were determined. Body surface area was calculated using the formula of DuBois.

### Blood pressure measurements

Resting brachial BP was measured before the exercise testing using a validated automatic device based on a standard sphygmomanometer technique (Boso clinicus, Bosch + Sohn GmbH & Co. KG, Germany). The cuff used for measurement was adjusted to the individual’s arm circumference. Measurements were performed by a trained research associate on both arms in a sitting position after a resting period of 5 min and repeated after 2 min. The average BP for each arm was calculated if both measurements were within 5 mmHg. The highest value was used for statistical analyses. Athletes with a resting SBP > 140 mmHg or diastolic BP (DBP) > 90 mmHg were excluded from the study.

### Exercise testing and assessment of maximum blood pressure

Athletes underwent a standardized progressive maximal cycling ergometer test with concurrent automatic brachial BP measurement and 12-lead ECG recording (Schiller AG^®^, Switzerland). The exercise test protocol for male athletes started with a load level of 100 W after a 2-min warm-up period that was conducted with 50 W. Female athletes started with 75 W after a warm-up period conducted with 50 W. Loads were increased by 50 W in male athletes and 25 W in females every 2 min until exhaustion, which was defined as the participant’s inability to maintain the load for 2 min. Next, the load was decreased to 25 W for 3 min of active recovery that was followed by a 2-min cool-down period at rest. The test concluded with a final ECG recording and brachial BP measurement. BP was measured once a minute during test and recovery periods, including at the maximum workload, immediately after the maximum workload, immediately after the end of the test, and after 5 min of recovery. BP was measured at the right arm during the test, and the participant was instructed to let the right arm hang loosely during measurement, when possible. SBP was recorded at the appearance of the first Korotkoff sound. Each BP measurement was protocolled automatically with the corresponding time, heart rate, and workload.

Heart rate was measured during continuous ECG recording throughout the test and recovery periods. We assessed the absolute maximum workload of all athletes as well as the respective relative workload, which was adjusted to individual body weight. Other measurements included maximum heart rate, heart rate at rest, and heart rate 5 min after the exercise test. Increases in systolic and diastolic BP were calculated from peak and baseline (resting) values. Pulse pressure was calculated as SBP minus diastolic blood pressure (DBP) at rest and at maximum exercise. In addition, mean brachial BP was determined as: DBP + (SBP − DBP)/3. MET values were estimated using the standard equations of the ACSM for cycling ergometers (Thompson et al. 2013). The ΔSBP was calculated as maximum SBP-resting SBP and was indexed by the increase in MET from rest (ΔMET calculated as peak MET − 1) to obtain the SBP/MET slope (Hedman et al. 2019). The peak SBP/W ratio was determined as peak SBP/peak workload in W (Hedman et al. 2020). The SBP/W slope was calculated as the ratio of the difference in SBP from the first to the last BP measurement during exercise divided by the difference in workload in W between these two measures (last SBP − first SBP)/(last W − first W) (Hedman et al. 2020).

### Non-invasive assessment of peripheral and central blood pressure and pulse pressure waveforms

We used the non-invasive vascassist2^®^ device (isymed GmbH, Butzbach, Germany) to acquire pulse pressure waveforms by means of oscillometry. The device uses a validated model (Schumacher et al. 2018) of the arterial tree that consists of 721 electronic circuits representing all central and peripheral arterial sections. By modulating the circuits’ capacitance, resistance, inductance, and voltage, the system replicates an individual’s acquired pulse pressure waves. The vascassist2^®^ system is currently unique in the use of genetic algorithms to optimize the fidelity of the of pulse pressure wave replication (Schumacher et al. 2018). Fidelity replications of 99.6% or above were included in the analysis.

The non-invasive vascular evaluation was carried out for all participants before exercise testing. After a 15-min rest period, measurements were performed in a supine position using four conventional cuffs adapted to the upper arm and forearm circumferences of the participants. Radial and brachial pulse pressure waves were acquired on both arms with step-by-step deflation of the cuffs. The measurements took place in a room with a comfortable and stable temperature of 22 °C and a lack of external stress influences. Participants were advised not to move during the acquisition of pulse pressure waves. Two brachial and three radial measurements were performed to guarantee stable and valid results with a break of 30 s between each measurement phase. The total duration of the examination was 15 min. The acquired pulse pressure waves were then analyzed with a validated electronic model of the arterial tree to assess vascular functional parameters. Brachial and radial SBP and DBP, central systolic and diastolic blood pressure (CBP), aortic pulse wave velocity (PWV), augmentation index (Aix), augmentation index at a heart rate of 75 bpm (Aix@75), resistance index (R), total vascular resistance, and ejection duration were calculated. CBP was determined using a transfer function that was based on the peripheral arterial waveform. Calculation of Aix@75 was also based on the pulse waveform.

### Statistical analysis

Descriptive analyses were carried out on all study variables for the total sample and separated by sex. All data are presented as mean ± standard deviation (SD). The Shapiro–Wilk test was used to determine normal distribution. If the data were determined to have a skewed distribution, all analyses were performed on normalized data. Between-group comparisons were made using independent sample t tests. Bivariate relations were analyzed using the Spearman correlation coefficient. Pearson’s product-moment correlation coefficient was used to determine linear correlations between vascular functional parameters and exercise test results. We also performed multivariate stepwise regression analyses to explore possible linear associations across the vascular functional parameters measured at rest in both sexes, using separately the SBP/MET slope, the peak SBP/W ratio and SBP/W slope as continuous dependent variable. Statistical significance was set at *p* < 0.05 (two-tailed) for all measurements. All statistical analyses were performed using the statistical software SPSS 25.0 for Mac (Statistical Package for the Social Sciences, Chicago, IL, USA).

## Results

### Cohort characteristics

A total of 72 participants, consisting of 47 male and 25 female athletes, were included in the study. Male athletes were taller and heavier and displayed a greater body mass index and a higher body surface area than females. Further, the training amount per week was higher than that of females. However, age (*p* = 0.365) and history of professional training were not different between groups (*p* = 0.112). The clinical characteristics, anthropometric data, and specific training data are displayed in detail in Table 1.

**Table 1:** Clinical characteristics of male (*n* = 47) and female (*n* = 25) athletes

	Male athletes *n* = 47		Female athletes *n* = 25		
	Mean	SD	Mean	SD	*p* value
Age (years)	21.6	1.7	21.1	2	0.365
Height (cm)	188.6	7.2	167.1	4.8	< 0.001
Weight (kg)	90.9	12.3	60.8	7.7	< 0.001
Body mass index (kg/m^2^)	25.5	2.4	21.7	1.9	< 0.001
Body surface area (m^2^)	2.18	0.17	1.68	0.11	< 0.001
Training history (years)	5.96	2.2	4.96	2.6	0.112
Training per week (hours)	17.85	2.9	10.8	2	< 0.001

Four of the examined 25 female athletes (16%) took oral contraceptives. The used hormonal contraceptives have a monophasic effect and are assigned to the fourth generation of hormonal contraceptives. They contained 3 mg drospirenone and 0.03 mg estradiol. In one case, the oral contraceptive contained 3 mg drospirenone and 0.02 mg estradiol. These combined oral contraceptive pills supply 21 days of pills with hormones followed by 7 days of hormone-free pills. This regimen is called the 21/7 regimen.

As expected, we found a significant correlation of age with history of professional training (*r* = 0.959, *p* < 0.001) in all athletes. Further, age was positively correlated with training per week (*r* = 0.226, *p* = 0.013), height (*r* = 0.298, *p* = 0.001), weight (*r* = 0.331, *p* < 0.001) and BMI (*r* = 0.314 *p* < 0.001). Height, weight, body surface area, and BMI all correlated positively with each other (*p* < 0.05) and with the training history and the training duration per week (*p* < 0.05).

### Blood pressure at rest

Resting brachial BP, mean brachial BP, and brachial pulse pressure at rest were not different between male and female athletes (*p* > 0.05). None of the participants displayed a BP > 140/90 mmHg. Further, heart rate at rest and central SBP were not different between the groups (*p* > 0.05). Male athletes had a significantly lower central diastolic BP (< 0.001) and mean CBP (*p* = 0.003) compared with female athletes. In contrast, the central pulse pressure (*p* < 0.001) was higher in males than in females. Detailed data are presented in Table 2.

**Table 2:** Results of vascular evaluation and exercise testing in male (*n* = 47) and female (*n* = 25) athletes

	Male athletes *n* = 47		Female athletes *n* = 25		
	Mean	SD	Mean	SD	*p* value
Brachial systolic BP (mmHg)	124.8	9.9	119.6	11.1	0.057
Brachial diastolic BP (mmHg)	75.7	7.3	73.5	8.4	0.284
Mean brachial BP (mmHg)	92.1	7	88.9	7.8	0.097
Pulse pressure at rest (mmHg)	49.1	9.2	46.1	11	0.248
Heart rate at rest (bpm)	57.2	10.2	61.9	10.2	0.068
Mean aortic blood pressure (mmHg)	72.4	8.9	79.3	8.9	**0.003**
Central systolic BP (mmHg)	97.7	8.3	95.7	8.5	0.340
Central diastolic BP (mmHg)	57.2	9.5	66.6	9.5	**< 0.001**
Central pulse pressure (mmHg)	37.2	6.5	28.9	4.7	**< 0.001**
Aortic pulse wave velocity (m/s)	6.2	0.85	5.9	0.58	0.079
Augmentation index @75 bpm (%)	− 20.7	11	− 17.4	9.6	0.203
Resistance index	16.15	6.5	12.7	10.7	0.148
Total vascular resistance (dyn*s/cm^5^)	1293	317.5	1218	341	0.369
Maximum heart rate (bpm)	179.3	11.8	183.5	7.8	0.079
Maximum systolic brachial BP (mmHg)	202.4	19.6	177.1	15.1	**< 0.001**
Δ systolic brachial BP (mmHg)	77.6	19.3	57.8	14	**< 0.001**
Maximum diastolic brachial BP (mmHg)	84.4	7.4	81.7	9.9	0.247
Δ diastolic brachial BP (mmHg)	8.7	9.5	8.2	8.6	0.821
Maximum pulse pressure (mmHg)	118	19.1	95.4	14.6	**< 0.001**
Absolute workload (Watt)	342	71.5	190	31.5	**< 0.001**
Relative workload (Watt/kg)	3.82	0.92	3.17	0.64	**< 0.001**
Peak energy expenditure (MET)	15.2	3.4	12.8	2.4	**0.001**
SBP/MET slope (mmHg/MET)	5.7	1.84	5.1	1.6	0.158
SBP/Watt slope (mmHg/Watt)	0.34	0.12	0.53	0.19	**< 0.001**
Peak SBP/Watt ratio (mmHg/Watt)	0.61	0.12	0.95	0.17	**< 0.001**

**Bold** text statistically significant differences

Resting SBP correlated positively with height, weight, BMI and sex. Resting DBP only correlated positively with age. Interestingly, central DBP was correlated with sex, but not central SBP. Further, central pulse pressure was correlated with sex.

### Vascular function at rest

The aortic pulse wave velocity (PWV) was not different between male and female athletes (*p* = 0.079). Further, the augmentation index at a heart rate of 75 bpm (Aix@75) (*p* = 0.203), the resistance index (R) (*p* = 0.148) and total vascular resistance (*p* = 0.369) were not different between the groups. In addition, none of the measured parameters of vascular function at rest were correlated to sex (*p* > 0.05). In contrast, PWV was correlated to age (*p* < 0.001) and BMI (*p* = 0.044), but not to height or weight (*p* > 0.05).

In male athletes, brachial systolic blood pressure was significantly correlated to the peak SBP/W ratio. Further, in male athletes, there was significant negative correlation of brachial diastolic blood pressure with the SBP/W slope. All other vascular functional parameters, measured at rest, were not significantly correlated to SBP/MET slope, the peak SBP/W ratio or the SBP/W slope in both male and female athletes. The results of the correlation analyses are presented in Table 3.

**Table 3:** Pearson’s correlations of the results of the vascular evaluation with the SBP/MET slope, the SBP/W slope and the peak SBP/W ratio for male and female athletes

	SBP/MET	SBP/W slope	peak SBP/W ratio
**Male athletes**			
Brachial systolic BP (mmHg)	0.073 (0.627)	0.025 (0.868)	**0.329 (0.024)**
Brachial diastolic BP (mmHg)	− 0.270 (0.067)	**− 0.342 (0.019)**	− 0.139 (0.350)
Mean aortic blood pressure (mmHg)	0.081 (0.589)	0.007 (0.964)	0.041 (0.787)
Central systolic BP (mmHg)	0.185 (0.214)	− 0.041 (0.783)	− 0.021 (0.888)
Central diastolic BP (mmHg)	− 0.055 (0.711)	− 0.128 (0.390)	− 0.076 (0.611)
Central pulse pressure (mmHg)	0.060 (0.669)	0.108 (0.469)	0.117 (0.432)
Aortic pulse wave velocity (m/s)	− 0.123 (0.412)	− 0.240 (0.104)	− 0.307 (0.063)
Augmentation index @75 bpm (%)	0.001 (0.996)	0.053 (0.725)	− 0.012 (0.934)
Total vascular resistance (dyn*s/cm^5^)	− 0.084 (0.575)	− 0.167 (0.262)	− 0.172 (0.248)
Resistance index	− 0.079 (0.596)	− 0.177 (0.233)	− 0.288 (0.058)

**Female athletes**			
Brachial systolic BP (mmHg)	− 0.239 (0.261)	− 0.125 (0.550)	− 0.162 (0.439)
Brachial diastolic BP (mmHg)	0.238 (0.262)	0.247 (0.234)	0.242 (0.244)
Mean aortic blood pressure (mmHg)	0.082 (0.703)	− 0.161 (0.443)	− 0.160 (0.445)
Central systolic BP (mmHg)	0.056 (0.794)	− 0.206 (0.322)	− 0.199 (0.340)
Central diastolic BP (mmHg)	0.109 (0.611)	− 0.143 (0.494)	− 0.143 (0.494)
Central pulse pressure (mmHg)	0.009 (0.966)	− 0.037 (0.861)	− 0.019 (0.928)
Aortic pulse wave velocity (m/s)	− 0.263 (0.204)	0.071 (0.735)	0.128 (0.543)
Augmentation index @75 bpm (%)	− 0.190 (0.374)	− 0.155 (0.458)	− 0.169 (0.420)
Total vascular resistance (dyn*s/cm^5^)	− 0.060 (0.782)	− 0.327 (0.111)	− 0.327 (0.111)
Resistance index	− 0.305 (0.147)	− 0.290 (0159)	− 0.308 (0.134)

**Bold** text signifies significant correlations

### Heart rate and blood pressure response to exercise

Male athletes displayed a significantly higher maximum SBP, higher ΔSBP and, thus, a higher maximum pulse pressure than female athletes. In contrast, maximum heart, maximum DBP, and ΔDBP were not different between males and females (Table 2). Maximum SBP, ΔSBP, and maximum pulse pressure were correlated with sex. In contrast, maximum DBP, ΔDBP, and maximum heart rate did not vary with sex.

### Performance and workload-indexed blood pressure responses

All participants completed the maximum exercise test until exhaustion, reaching a maximum heart rate above the calculated individual 85% threshold (of individually calculated maximum heart rate). Male athletes achieved a significantly higher absolute workload than female athletes with a correspondingly higher relative workload and MET.

The SBP/W slope (0.34 ± 0.12 vs. 0.53 ± 0.19 mmHg/W, *p* < 0.001) and the peak SBP/W ratio (0.61 ± 0.12 vs. 0.95 ± 0.17 mmHg/W, *p* < 0.001) were significantly lower in male athletes than in female athletes. However, the SBP/MET slope was not different between males and females (5.7 ± 1.84 vs. 5.1 ± 1.6 mmHg/MET, *p* = 0.158) (Table 2).

The SBP/W slope (*r* = 0.633, *p* < 0.001) and the peak SBP/W ratio (*r* = 0.761, *p* < 0.001) were correlated with sex, but the SBP/MET slope (*r* = − 0.162, *p* = 0.178) was not.

The SBP/MET slope was positively correlated with height (*r* = 0.257, *p* = 0.030), weight (*r* = 0.326, *p* = 0.006), and BMI (0.322, *p* = 0.006) but not with age (*r* = 0.077, *p* = 0.407).

In contrast, the SBP/W slope was negatively correlated with age (*r* = − 0.233, *p* = 0.010), height (*r* = − 0.573, *p* < 0.001), weight (*r* = − 0.571, *p* < 0.001), and BMI (*r* = − 0.459, *p* < 0.001).

Further, negative correlations of the peak SBP/W ratio with height (*r* = − 0.691, *p* < 0.001), weight (*r* = 0.691, *p* < 0.001), and BMI (− 0.483, *p* < 0.001) were found.

The SBP/MET slope was the only workload-indexed marker of BPR that was not correlated with both age and sex.

### Regression analyses of the influence of the hemodynamic data of athletes on different markers of workload-indexed blood pressure response

We performed multivariate regression analyses to explore possible linear associations across the vascular functional parameters measured at rest in both sexes with the workload-indexed markers of BPR. We used brachial systolic BP, brachial diastolic BP, central systolic and diastolic BP, mean central BP, central pulse pressure, PWV, Aix@75 bpm, R and total vascular resistance as predictors of the regression model and, separately, the SBP/MET slope, the peak SBP/W ratio and SBP/W slope as continuous dependent variable in both sexes. All evaluated regression models were not able to predict the markers of workload-indexed BPR in both sexes and neither of the evaluated vascular functional parameters at rest were found to be independent determinants of the workload-indexed BPR. Details of the respective regression analyses are presented in Table 4.

**Table 4:** Stepwise multivariate regression analyses of the results of the vascular evaluation with the SBP/MET slope, the SBP/W slope and the peak SBP/W ratio as continuous dependent variable for male and female athletes

	Male athletes			Female athletes		
	*R*^2^	*F*	*p* value	*R*^2^	*F*	*p* value
SBP/MET slope	0.161	*F*(8, 38) = 2.101	0.060	− 0.214	F(8, 15) = 0.493	0.843
SBP/W slope	0.139	*F*(8, 38) = 1.928	0.084	0.130	*F*(8, 16) = 0.656	0.722
Peak SBP/W ratio	0.170	*F*(10, 36) = 1.942	0.071	− 0.114	*F*(10, 14) = 0.754	0.668

Predictors of the model were brachial systolic BP, brachial diastolic BP, central systolic and diastolic BP, mean central BP, central pulse pressure, PWV, Aix@75 bpm, R and total vascular resistance

## Discussion

The present study is, to our knowledge, the first to investigate sex differences in markers of workload-indexed BPR and their correlations with parameters of vascular function in age-matched professional athletes.

Our most important findings are that,

1. The SBP/W slope and the peak SBP/W ratio were significantly different between female and male athletes whereas the SBP/MET-slope was not different.
2. The SBP/W slope and peak SBP/W ratio was significantly higher in females despite males displaying a significantly higher maximum SBP.
3. None of the parameters of vascular function at rest predicted these gender-based differences.

These findings imply different physiological adaptations of BPR to exercise between females and males that are not revealed with the measurement of central hemodynamics and vascular function at rest.

In general, given different exercise testing methods (Weiss et al. 2010; Jae et al. 2015), BP measurement methods (Weiss et al. 2010; Hedman et al. 2019) and determinations of SBP at maximum (Hedman et al. 2019; Bauer et al. 2020; Pressler et al. 2018; Caselli et al. 2016) or submaximal (Weiss et al. 2010) workload, a comparison across studies is challenging. Further, studies investigating the BPR to exercise in athletes are sparse (Pressler et al. 2018; Caselli et al. 2016) and workload-indexed data for female athletes are not available, so far.

Our study provides the first comparison of the newly introduced markers of workload-indexed BPR to exercise in male and female professional athletes.

Recently, Hedman et al. (2020) reported age- and sex-specific reference equations for workload-indexed systolic BPR during bicycle ergometry for the general population.

The SBP/W slope, reflecting the increase of SBP per W increment and thus the steepness of SBP in relation to workload, was reported to be markedly higher in females than in males (Hedman et al. 2020), indicating a different physiological adaptation of SBP to exercise (Smith et al. 2019; Wheatley et al. 2014; Hedman et al. 2020). In line with this, female athletes of our study displayed a significant higher SBP/W slope compared to male athletes.

These sex differences in the SBP/W slope indicates women’s need for a larger relative increase in cardiac output to generate the same power output (Joyner et al. 2016; Wheatley et al. 2014). In consequence, lower achieved absolute and relative workloads in women were partly explained with different body composition, especially lower lean muscle mass compared to men (Wheatley et al. 2014; Joyner et al. 2016; Song et al. 2020; Samora et al. 2019).

Another interesting influencing factor that attributes to the different BPR in women compared to men was identified with different metaboreceptor stimulation in women (Samora et al. 2019). Notably, in pre-menopausal women, beta-adrenergic sensitivity is enhanced compared to men which blunts vasoconstrictor response due to concurrent beta-adrenergic mediated vasodilation (Song et al. 2020) during constant-load submaximal exercise and consequently leads to a limitation in stroke volume (Wheatley et al. 2014).

Though, at the highest exercise intensities, a metaboreflex-driven increase in peripheral resistance is observed (Augustyniak et al. 2001; Smith et al. 2019), which may be altered in trained women compared to untrained women. These deliberations might partly explain the differences in the SBP/W slope of our female athletes (0.53 ± 0.19 mmHg/W) compared to the proposed normative 50th percentile of 0.38 ± 0.14 mmHg/W of the Hedman cohort (Hedman et al. 2020). Hence, it can be speculated that higher fitness levels, and thus enhanced abilities to maintain physical performance, contribute to a higher SBP/W slope in athletic women.

In contrast and in line with the aforementioned, the BPR to exercise in men is characterized by an increase in cardiac output and by an increase in total peripheral resistance (Samora et al. 2019) and therefore less influenced by fitness levels. Hence, the values of our male athletes were comparable (0.34 ± 0.12 vs. 0.33 ± 0.11 mmHg/W) to the proposed reference values of the general population.

These findings of sex differences in BPR to exercise in both trained and untrained individuals indicate a different BP regulation during exercise between men and women that is irrespective of fitness levels, complex and not yet fully understood (Samora et al. 2019; Joyner et al. 2016).

Further, these considerations might also explain the sex differences in the peak SBP/W ratio, which were described by Hedman et al. (2020) and even were apparent in our study cohort of professional athletes. The peak SBP/W ratio showed markedly higher values in women of both studies. The larger peak SBP/W ratio at a lower absolute peak SBP in women compared to men hints at men’s capacity to extract a relatively large increase in power output (once cardiac reserve for cardiac output has been reached) from the exercise pressor reflex-generated increase in the sympathetic outflow that increases perfusion pressure by increasing total vascular resistance.

In line with the aforementioned, the absolute values of the peak SBP/W ratio in our cohort of professional athletes were lower compared to the published reference values (Hedman et al. 2020). Given that our athletes had significantly greater cardiorespiratory fitness (CRF) than the participants of the Hedman study (3.82 W/kg vs. 2.8 W/kg and 3.17 W/kg vs. 2.08 W/kg for males and females, respectively) it is tempting to speculate that CRF affects the SBP/W slope differently in men and women. Hence, the peak SBP/W ratio of athletes might be lower compared to that of controls, as athletes usually achieve higher workloads. In consequence, these results raise the question whether athletes might need different thresholds for the peak SBP/Watt ratio. Thus far, the presented reference values of the peak SBP/W ratio for the general population should not be used as the only workload-indexed marker of BPR in the interpretation of exercise testing of professional athletes of both sexes.

In contrast to the SBP/W slope and the peak SBP/W ratio, we could not detect significant sex differences in the SBP/MET slope.

Unlike the SBP/W slope and the peak SBP/W ratio, the SBP/MET slope is unaffected by bodyweight as METs are corrected for body weight. Hence, division of the change of SBP by this ratio informs about the rate of blood pressure change relative to an increase of exercise intensity above the resting baseline. This explains why there was no significant difference in SBP/MET slope between our female and male athletes (5.1 mmHg vs. 5.7 mmHg in females and males, respectively). In another study a median of 6.4 mmHg/MET in a male cohort of a general population (Hedman et al. 2019) was reported, indicating that the proposed threshold of 10 mmHg/MET (Thompson et al. 2013) represents an upper limit rather than an average normal increase. Considering that a higher SBP/MET slope (> 6.2 mmHg/MET) was associated with worse survival in a normal population of male non-athletes (Hedman et al. 2019), the proposed SBP/MET slope might help to identify individuals at risk, which would be crucial for preventive interventions.

Given its apparent independence of gender the SBP/MET slope may be already used for the interpretation of the BPR to exercise in athletes of both sexes during the pre-participation screening. Notably, the SBP/MET slope was the only evaluated workload-indexed parameter of our study that was not different between male and female athletes.

Thus, the proposed normative values were derived from 285 males and 97 females in the Hedman cohort (Hedman et al. 2020), and the prognostic value of the SBP/W slope, the peak SBP/W ratio and the SBP/MET slope needs to be confirmed in the future.

As described above, the complex and ambiguous association between the BPR to exercise and cardiovascular risk might be explained with vascular function and vascular adaptation to exercise (Green et al. 2012). Further, increased arterial stiffness was related to an exaggerated BPR in a general population (Thanassoulis et al. 2012), and PWV was able to predict the BPR to exercise in healthy young adults (Haarala et al. 2020). Thus, in our cohort of highly trained male and female athletes, we did not detect differences, despite our attempt to minimize potentially confounding influences of the menstrual cycle.

Hence, the influence of oral contraceptives (OCP) of the fourth generation that were taken by four athletes seems unlikely, as these OCP are currently seen not to affect peripheral vasculature (Williams and MacDonald 2020) or BP negatively (Ribeiro et al. 2018).

Another confounding factor that may have influenced the measurements of PWV and BP are postprandial effects, especially raised serum triglycerides and lipemia. Thus far, previous studies concerning this topic have yielded inconsistent results (Taylor et al. 2014; Lithander et al. 2013) and data for professional athletes are sparse. In healthy young individuals (mean age 25.6 years) no measureable increase in PWV after a mixed meal high in saturated fat were found (Taylor et al. 2014), indicating that postprandial effects are unlikely to influence our findings.

The PWV we determined in our athletes is in line with other studies that investigated athletes (Vlachopoulos et al. 2010; Bauer et al. 2019) and with recent meta-analyses (Ashor et al. 2014). Global vascular resistance at rest, R, and PWV at rest were not different between male and female professional athletes in our current study, and, consequently, they were not able to predict the workload-indexed markers of BPR. In addition, all central hemodynamic parameters, determined at rest, did not correlate with the workload-indexed markers of BPR and were not able to predict them.

In conclusion, it may be speculated that resistance index and global vascular resistance differ during exercise conditions and lead to the detected difference in the workload-indexed BPR with male athletes displaying a higher arterial vasodilator reserve compared to women. Unfortunately, vascular resistance could not be measured during exercise to substantiate this hypothesis. Taken together, these results highlight the problems inherent to the use of non-invasive devices that evaluate vascular function via oscillometry (Miyata 2018). These validated methods deliver reliable results at rest, but not during an exhaustive exercise test (Miyata 2018).

Our study has attempted to identify reliable sex differences in vascular functional parameters at rest to predict the workload-indexed BPR to a maximum exercise test. However, this was not the case, and even PWV was not different. As total vascular resistance at rest and SBP were not different between male and female athletes, our data indicate the importance of the different vascular functional regulation during exercise for the BPR to exercise in both sexes.

### Limitations and strengths

Our study has a few limitations. The number of participants limited its power to uncover potential correlations between the workload-indexed BPR parameters measured and markers of cardiac and vascular function other than CBP, PWV, total vascular resistance, brachial BP, and the BPR to a maximum exercise test. The focus on professional soccer and handball athletes may limit extrapolation of the results to other sport disciplines; however, as these team sports expose athletes to the hemodynamic stress of frequent interval sprints, it is representative of other sports with a high dynamic component (> 75% *V*O_2max_) and a moderate static component (10–20%) (Levine et al. 2015). Another limitation is the difference in training hours per week between males and females that may have influenced the results. Thus, female athletes in team sports usually display lower training volumes per week than their male peers. Further, we did not control for diet and potential postprandial effects, body composition, and ventricular function. Our exclusive focus on young male and female athletes precludes the extrapolation of our results to athletes of other age groups. However, we included professional male and female athletes of the same age without cardiovascular disease and free of medication, and we controlled for the menstrual cycle to minimize confounders. Thus, four female athletes were taking oral contraceptives of the fourth generation, which might have influenced the BP. Further, the rigid design of measuring vascular function and accomplishing an exhaustive and standardized exercise test in both sexes must be mentioned. Therefore, our cohort of professional handball and soccer athletes, although small, was homogeneous, which strengthens our analysis.

## Conclusion

In our cohort of professional athletes, we detected sex differences in the SBP/W slope and the peak SBP/W ratio, with a steeper BP increase in females. In contrast, we identified the SBP/MET slope as a sex-independent marker of workload-indexed BPR. Despite sex differences in central hemodynamics and vascular function measured at rest, these parameters were not able to predict any of the workload-indexed markers of BPR in males and females. These findings emphasize the link between vascular function, total vascular resistance, BPR, and physical performance during exercise in athletes. Further, a sex-specific consideration of BPR to exercise testing, which is frequently performed in the cardiovascular evaluation of competitive athletes, is encouraged to identify athletes at risk.

## Abbreviations

ACSM: American College of Sports Medicine
Aix@75 bpm: Augmentation index at a heart rate of 75 bpm
BP: Blood pressure
BPR: Blood pressure response
CBP: Central blood pressure
DBP: Diastolic blood pressure
ECG: Electrocardiogram
MET: Metabolic equivalent of task
Peak SBP/W ratio: Ratio of systolic blood pressure and W at peak exercise
PWV: Pulse wave velocity
SBP: Systolic blood pressure
SD: Standard deviation
SBP/MET slope: Increase in systolic blood pressure per increase in metabolic equivalent of task
SBP/W slope: Increase in systolic blood pressure per increase in W
*V*O_2max_: Maximum oxygen uptake
